# Persistent microbial infections and idiopathic pulmonary fibrosis - an insight into *non-typeable Haemophilus influenza* pathogenesis

**DOI:** 10.3389/fcimb.2024.1479801

**Published:** 2024-12-20

**Authors:** Anthony Shadid, Haydn E. Rich, Hunter DeVaughn, Aleksey Domozhirov, Marie- Françoise Doursout, Tingting Weng-Mills, Kristin L. Eckel-Mahan, Harry Karmouty-Quintana, Marcos I. Restrepo, Pooja Shivshankar

**Affiliations:** ^1^ Center for Metabolic and Degenerative Diseases, The Brown Foundation Institute of Molecular Medicine for Prevention of Human Diseases, UTHealth-McGovern Medical School, Houston, TX, United States; ^2^ Department of Biochemistry and Molecular Biology, UTHealth-McGovern Medical School, Houston, TX, United States; ^3^ Department of Anesthesiology, Critical Care and Pain Medicine, UTHealth-McGovern Medical School, Houston, TX, United States; ^4^ Division of Pulmonary and Critical Care Medicine, Department of Medicine, South Texas Veterans Health Care System and the University of Texas Health San Antonio, San Antonio, TX, United States

**Keywords:** interstitial lung diseases (ILD), persistent microbial infection, idiopathic pulmonary fibrosis (IPF), *non-typeable-Haemophilus influenzae* (*NTHi*), clinical studies in IPF, mRNA-based therapeutics targeting intracellular pathogens

## Abstract

Interstitial lung disease (ILD) is characterized by chronic inflammation and scarring of the lungs, of which idiopathic pulmonary fibrosis (IPF) is the most devastating pathologic form. Idiopathic pulmonary fibrosis pathogenesis leads to loss of lung function and eventual death in 50% of patients, making it the leading cause of ILD-associated mortality worldwide. Persistent and subclinical microbial infections are implicated in the acute exacerbation of chronic lung diseases. However, while epidemiological studies have highlighted pollutants, gastric aspirate, and microbial infections as major causes for the progression and exacerbation of IPF, the role of persistent microbial infections in the pathogenesis of IPF remains unclear. In this review, we have focused on the role of persistent microbial infections, including viral, bacterial, and fungal infections, and their mechanisms of action in the pathogenesis of IPF. In particular, the mechanisms and pathogenesis of the Gram-negative bacteria Non-typeable *Haemophilus influenzae* (*NTHi*) in ILDs are discussed, along with growing evidence of its role in IPF, given its unique ability to establish persistent intracellular infections by leveraging its non-capsulated nature to evade host defenses. While antibiotic treatments are presumably beneficial to target the extracellular, interstitial, and systemic burden of pathogens, their effects are significantly reduced in combating pathogens that reside in the intracellular compartments. The review also includes recent clinical trials, which center on combinatorial treatments involving antimicrobials and immunosuppressants, along with antifibrotic drugs that help mitigate disease progression in IPF patients. Finally, future directions focus on mRNA-based therapeutics, given their demonstrated effectiveness across a wide range of clinical applications and feasibility in targeting intracellular pathogens.

## 1 Introduction

**Figure 1:**
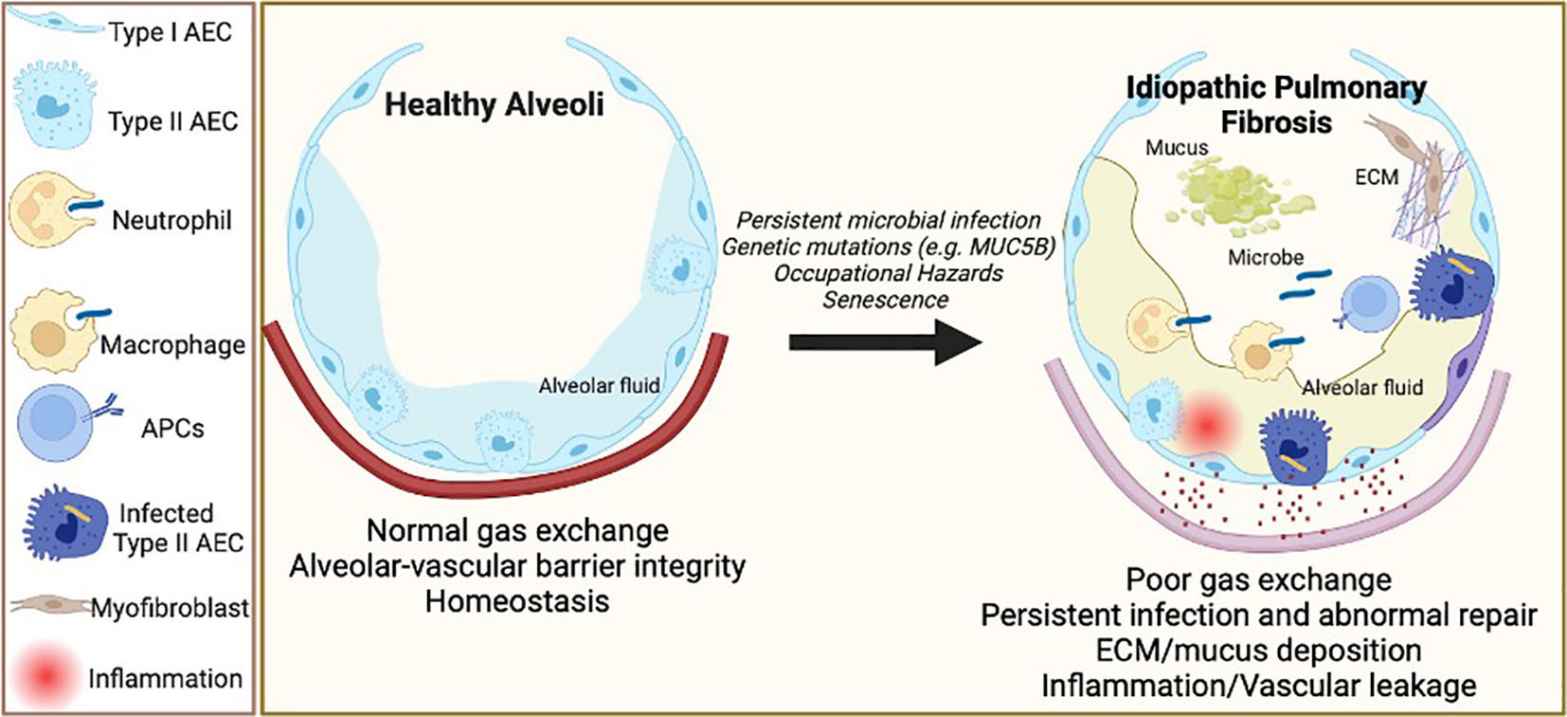
Pathogenesis of Idiopathic Pulmonary Fibrosis (IPF) and microbial persistence. The etiology of IPF includes a multitude of triggers to the homeostatic lung functioning. Occupational and chronic exposure to particulate matter, allergens, persistent microbial infections, age-associated inflammation and senescence, and genetic predisposition lead to repetitive alveolar injury and increase the risk of developing IPF. Alveolar epithelial cells (AEC) play a central role in maintaining the alveolar-vascular barrier and initiating repair after injury, while antigen-presenting cells (APC) mediate immune responses to microbial infections. Compared to healthy lungs with normal gas exchange and alveolar-vascular barrier integrity, persistent microbial infection and intracellular inhabitance may lead to abnormal alveolar repair with vascular leakage and uncontrolled inflammation. These aberrant alveolar/vascular functions further lead to mucus deposition and fibroblast activation to produce extracellular matrix (ECM) components, causing scarring of the lungs and poor gas exchange, thereby declining the overall lung function.

Idiopathic Pulmonary Fibrosis (IPF) is an idiopathic, progressive, and chronic illness that causes an accumulation of scarred tissue in the lungs, leading to difficulty breathing and disseminating oxygen into the bloodstream. Idiopathic pulmonary fibrosis is typically seen in people over the age of 50 (Lederer and Martinez, 2018). In the United States, the adjusted prevalence is estimated to be 2.4 cases per 10,000 people (Nalysnyk et al., 2012; Maher et al., 2021). The prognosis is poor, and most individuals only survive 2-3 years following diagnosis (Strongman et al., 2018). The majority of IPF cases are sporadic and occur with no prior family history of this disease. However, there are still instances of familial pulmonary fibrosis, which is believed to exhibit a pattern of autosomal dominant inheritance, wherein a single copy of the altered gene is sufficient to cause the illness. Even then, there are individuals who, despite inheriting the altered gene, do not go on to develop fibrosis. The reasons for this remain unclear. Occupational exposures can also increase the likelihood of developing idiopathic pulmonary fibrosis; farming, agricultural professions, and pesticide contact raise the risk of IPF development (**Figure 1**). Smoking and past exposure to metal or wood dust are also associated with higher rates of IPF (Paolocci et al., 2018; Park et al., 2021).

Overall, 20% to 50% of all ILDs exhibit clinical manifestations of IPF, presenting unique histological phenotypes, including usual interstitial pneumonia, areas of honeycombing, and fibroblastic foci. To date, two antifibrotic therapies that have been FDA-approved and are available to treat IPF include Pirfenidone and Nintedanib. Studies have shown that Pirfenidone and Nintedanib effectively inhibit fibroblast proliferation and activity and the deposition of extracellular matrix proteins in the lungs, both critical processes in developing lung fibrosis (Conte et al., 2014; Wollin et al., 2015). To that end, these drugs indeed work to slow the progression of the disease. However, they do not reverse the fibrotic damage that has already occurred, nor do they completely halt disease progression. Clinical studies and real-world data suggest that both Pirfenidone and Nintedanib offer modest improvements in lung function and survival but fall short of providing a cure. Their efficacy is limited to reducing the rate of decline in lung function. Moreover, both drugs are associated with significant side effects, including gastrointestinal disturbances, liver dysfunction, nausea, and fatigue, which can impact patients’ quality of life and lead to treatment discontinuation (Finnerty et al., 2021; Kou et al., 2024). This underscores the need for more effective therapies that not only slow disease progression but also reverse existing fibrosis and improve overall outcomes for patients.

Furthermore, abundant research has studied associations between IPF and viruses, such as human metapneumovirus, influenza virus, and coronaviruses. Idiopathic pulmonary fibrosis patients are coinfected with viral and bacterial infections show significantly diminished lung function and increased risk of mortality (Moghoofei et al., 2022). However, the contributions of persistent bacterial infections in IPF are understudied. The main body of this review seeks to elucidate connections between these infections and specific mechanisms utilized by their causative pathogens, which may accelerate or complement fibrotic processes in IPF. It examines the role of Gram-positive and Gram-negative bacteria infections and highlights promising avenues for future investigation into possible treatments and diagnostic methods.

Here, we discuss how viral, fungal, and bacterial infections contribute to the pathogenesis of IPF, review the literature, and identify areas where further investigation is necessary, with an emphasis on the commensal Gram-negative bacterium *Haemophilus influenzae*. It is among the most common causes of upper and lower respiratory tract infections in adults and children (Ieven et al., 2018; Lv et al., 2024). The non-typeable *Haemophilus influenzae* (*NTHi*) exacerbates the pathogenesis of chronic obstructive pulmonary disease (COPD), airway inflammation, and end-stage lung diseases (Su et al., 2018; Saliu et al., 2021). For example, overactivity of the mucin-producing gene *MUC5B* is associated with goblet cell dysfunction in COPD and IPF (Molyneaux et al., 2014; Hanmandlu et al., 2022; Huang et al., 2022). Chronic middle ear infection with *NTHi* is also associated with dysregulation of *MUC5B* mucins (Val et al., 2015). However, the mechanisms of *NTHi* intracellular pathogenicity are underexplored in the progression of lung diseases. As for COPD, we suggest here that persistent intracellular *NTHi* infection should be investigated in the context of progressive IPF development.

Altogether, this review addresses a critical gap in the literature, as no recent or comprehensive analyses have thoroughly examined this specific topic. Unlike a systematic review, which emphasizes clinical research and methodology, this work offers an in-depth perspective on mechanistic insights and the underlying processes that could intrigue future therapeutic strategies.

## 2 Viral Infections in IPF

Numerous studies have shown that viral infections may play a key role in the pathogenesis of IPF (Sheng et al., 2020). Most of these studies have looked explicitly at Epstein-Barr virus (EBV) and other herpesviruses in relation to IPF. In a serological study, Manika et al. reported that 60% of patients with IPF had anti-EBV immunoglobulin A (IgA) (*P*=0.024), compared to only 22% of control patients (Manika et al., 2007). Additionally, the group found EBV DNA via polymerase chain reaction (PCR) in the bronchoalveolar lavage (BAL) fluid of 3 of 17 patients with IPF. A study by Kelly et al. investigated the presence of WZhet, a rearranged genetic frame of EBV associated with productive EBV replication, in both IPF patients and controls (Kelly et al., 2002). Despite 75-85% of patients across all groups testing positive for EBV DNA by buffy coat analysis, 0% and 4% of the two control groups were positive for WZhet, whereas 59% of IPF patients had WZhet present in peripheral blood samples. Since 61% of EBV-positive lung biopsies in IPF patients also demonstrated WZhet presence, evidence indicates a correlation between WZhet expression in lung tissue and peripheral blood. Despite broad evidence for the role of immunosuppressive therapy in reactivating herpes viruses, this study noted no relationship between prior immunosuppressive treatments and WZhet expression. These data further confirm the association between active EBV infection and IPF, suggesting a potential marker in the peripheral blood for tracking EBV in this disease. Numerous studies have reported the presence of viral DNA in IPF patients, such as the detection of EBV by PCR and immunohistochemistry in 11/27 of patients with IPF but not in controls (Stewart et al., 1999). Furthermore, *Herpesvirus saimiri* DNA, a virus naturally found in squirrel monkeys, was also detected in the regenerating epithelial cells of 21/21 IPF biopsy samples examined (Folcik et al., 2014). The latter case is of particular interest, given that *H. saimiri*’s low infection rate in humans (around 7%) aligns better with its potential role as an etiological factor in a rare disease like sporadic IPF (Moore and Moore, 2015). Murine gammaherpesvirus-68 (MHV-68) also shares significant homology with *H. saimiri*, and it is a widely used strain for preclinical studies on the effects of herpesvirus on lung fibrosis (Bortz et al., 2003; Folcik et al., 2014). Altogether, these studies point to herpesviruses as potentially significant in the pathogenesis of IPF.

Among the first studies to propose a mechanism for the viral pathogenesis of IPF is one that investigates the role of malfunctioning type II alveolar epithelial cells (AECs) in IPF patients, showing the presence of herpes virus protein (Lawson et al., 2008). Specifically, the group investigated the role of surfactant protein C (SFTPC) mutations in IPF, examining how mutant SFTPC expression may induce endoplasmic reticulum (ER) stress and the unfolded protein response (UPR) in AECs. They found herpesvirus proteins in AECs from 15/23 IPF patients, which colocalized with UPR marker XBP-1 (X-box binding protein-1), indicating a potential link between herpesvirus infection, ER stress, and IPF progression (Lawson et al., 2008). This suggests that ER stress and UPR activation in the alveolar epithelium could contribute to the development and worsening of IPF, possibly through chronic herpesvirus infection or altered surfactant protein processing. Systemic sclerosis-associated IPF patients also showed qPCR positivity results for the presence of EBV latent membrane protein-1 (LMP1) that could be associated with more rapid disease progression in IPF. In a one-year follow-up study, it was reported that a large number of LMP1-positive patients with IPF died from respiratory failure as compared to LMP1-negative patients, suggesting that EBV LMP1 could play a role in the progression of IPF (Tsukamoto et al., 2000). A case study reinforced these findings, indicating that latent LMP1-positivity correlated with poor prognosis (Marzouk et al., 2005). Idiopathic pulmonary fibrosis patients also demonstrated EBV and p53 expression via immunohistochemistry, compared to the absence of expression in the control group (*P* = 0.05). This suggested that a functional relationship between EBV and p53 may exist in patients with IPF, as p53 activity is central in regulating the cell cycle and apoptotic cell death (Lok et al., 2001). Intriguingly, administering the antiviral medication Ganciclovir to advanced IPF patients with signs of previous EBV showed promising results, weakening the progression of IPF (Egan et al., 2011).

Preclinical studies have helped us gain deeper insights into the mechanisms by which a gamma herpesvirus (γHV) infection can influence the development of lung fibrosis. The first example of herpesvirus-inducing fibrosis in a natural host came from a study by Williams et al., who showed that infecting horses with an equine γHV led to the development of fibrosis (Williams et al., 2013). In murine models, prior infection with MHV-68, even in its latent state, can amplify lung fibrosis triggered by subsequent fibrotic insults with bleomycin or fluorescein isothiocyanate (Vannella et al., 2010). This augmentation of fibrosis seems unrelated to active viral replication. Moreover, latent viral infection in mouse lungs correlates with heightened production of chemokines attracting fibrocytes and inflammatory cells and increased release of cysteinyl leukotrienes, tumor necrosis factor α, and profibrotic transforming growth factor β1 (TGF-β1), suggesting potential profibrotic mechanisms involved in viral influence on pulmonary fibrosis (Vannella et al., 2010; Stoolman et al., 2011). While these studies have demonstrated that prior infections may increase susceptibility to subsequent fibrotic injury by altering lung epithelial cells during latency and prompting their secretion of profibrotic factors, viral infection can also exacerbate fibrotic disease when superimposed on pre-established fibrosis (McMillan et al., 2008). In such cases, active lytic replication correlates with increased TGF-β1 signaling and epithelial cell apoptosis, ultimately resulting in increased collagen deposition within the lung. Notably, not all viral infections cause the same degree of fibrosis in the lung. For example, MHV-68, but not influenza A (H1N1), was able to exacerbate lung fibrosis in mice, indicating there may be some specificity involved (Ashley et al., 2014).

Several studies have investigated the role of other viruses in IPF, although most have found little to no association. For instance, despite adenovirus’ capacity to stimulate the secretion of TGF-β1 from epithelial cells and induce epithelial-mesenchymal transition (EMT) via its E1A protein, adenovirus activity has ultimately shown limited correlation with IPF (Hayashi and Hogg, 2007). Similarly, the prevalence of adenovirus, enterovirus, or bocavirus DNA in lung biopsy samples, nasopharyngeal swabs, and bronchioalveolar lavage (BAL) fluids did not correlate significantly with the induction of profibrotic transformation in IPF patients compared to controls (Kuwano et al., 1997; Moradi et al., 2017).

The hepatitis C virus (HCV), which is known to trigger liver cirrhosis with chronic infection, has also been explored in the studies of lung fibrosis. A positive association between HCV and IPF was observed, with increased HCV antibody levels detected in IPF patients compared to controls (Ueda et al., 1992; Zidan et al., 2015), and increased incidence of IPF in patients previously infected with HCV compared to those infected with hepatitis B virus (Arase et al., 2008). Elevated anti-HCV antibody levels were also shown to be consistent across other lung diseases, suggesting it may not be exclusive to IPF (Irving et al., 1993; Meliconi et al., 1996).

In a 2001 study testing the sera of 33 IPF patients, the recently identified Torque teno virus (TTV) was present in 36.4% of patients. Additionally, the TTV-positive group exhibited a significantly worse 3-year survival rate (58.3%) than the TTV-negative group (95.2%) (Bando et al., 2001). Another study found that TTV was the most common virus in patients with acute exacerbation of IPF and was also present in patients with acute lung injury (Huie et al., 2010; Wootton et al., 2011). However, another study measuring TTV DNA titers in patients with acute exacerbation of IPF suggested that an association between TTV and the onset of acute exacerbation of IPF was unlikely (Bando et al., 2015).

Furthermore, the proposed mechanism of SARS-CoV-2-induced pulmonary fibrosis has striking similarities to fibrotic processes observed in IPF. The virus damages alveolar epithelial cells type 2 (AEC2), inciting macrophage activation and causing additional injury to the alveolar basement membrane. Macrophage response and AEC2 injury precipitate the release of inflammatory regulators such as IL-6, TNF-α, and TGF-β1. However, it is TGF-β1 that primarily acts to encourage fibroblast proliferation and differentiation into myofibroblasts (Alrajhi, 2023; Duong-Quy et al., 2023). Patients with IPF also have increased pulmonary levels of TGF-β1, and TGF-β1-mediated stimulation of the epithelial-to-mesenchymal transition is widely believed to contribute to progressive fibrosis (Wolters et al., 2014). Current studies center on the effectiveness of antifibrotic agents classically used for IPF in treating post-COVID-19 pulmonary fibrosis (Patrucco et al., 2023).

Nonetheless, host antiviral mechanisms such as the mitochondrial antiviral signaling protein (MAVS) may also contribute to the pathogenesis of IPF. In bleomycin-induced pulmonary fibrosis, mice showed increased MAVS response to damage-associated molecular patterns (DAMPs) generated by bleomycin injury. By mimicking the BH3 components, MAVS- downstream targets B-cell lymphoma-2 complex (Bcl-2), and Bcl-xl pro-apoptotic proteins attenuated MAVS-mediated fibrotic pathology (Kim et al., 2021). It is noteworthy that Bcl-2 and Bcl-xl complex also mediate anti-apoptotic and anti-proliferative effects as a unique dual-cell cycle response that may be responsible for the BH3 mimetic-mediated attenuation of fibrosis (Janumyan et al., 2003). Strikingly, the SARS-CoV2 nucleocapsid protein activates the Bcl-2 family protein, myeloid-cell leukemia-1 protein (MCL-1), to inhibit apoptosis, enhancing viral propagation and infectivity (Pan et al., 2023). In contrast, SARS-CoV2 manipulates MAVS signaling by several non-specific proteins from the open reading frames ORF3a, ORF9, and ORF10, thereby leading to increased lung tissue injury and non-resolvable COVID-19 interstitial lung disease (Wu et al., 2021; Wang et al., 2022). Another antiviral response protein, the engulfment and motility (ELMO) domain containing-2 protein (ELMOD-2), is also genetically implicated in IPF pathology. The antiviral mechanisms of ELMOD-3 were elucidated using the overexpression and knockdown strategies in A549 alveolar epithelial cells and demonstrated to be mediated by intracellular TLR3 signaling during influenza viral infections (Hodgson et al., 2006; Pulkkinen et al., 2010). Interestingly, SARS-COV2 infection seems to downregulate the expression of ELMOD2 and, therefore, ELMOD-2-mediated antiviral response (Radzikowska et al., 2023).

While there certainly are similarities in fibrotic pathways between IPF and SARS-CoV-2-related pulmonary fibrosis, it is critical to recognize their distinct clinical trajectories. SARS-CoV-2 infection is known to induce fibrotic changes, often evident on CT scans, particularly in patients with severe respiratory illness. However, longitudinal studies have demonstrated that these fibrotic changes largely resolve over time in the majority of patients, especially those with mild-to-moderate disease (Ahamed et al., 2024; Cortes-Telles and Zavorsky, 2024; Pinto et al., 2024). Unlike IPF, where fibrosis progresses inexorably and irreversibly, post-COVID-19 fibrosis appears transient and more reflective of reparative mechanisms following acute lung injury.

The differences in outcomes are underpinned by diverging pathophysiological processes. Idiopathic pulmonary fibrosis is characterized by chronic, progressive scarring driven by genetic predispositions, environmental exposures, and dysregulated fibroblast activity. In contrast, SARS-CoV-2-related fibrosis is primarily an acute inflammatory response to alveolar epithelial damage, often mediated by cytokine storms and endothelial injury. Shared pathways, such as TGF-β1 signaling and epithelial-to-mesenchymal transition, are activated in both conditions; however, their duration and impact differ significantly. For example, TGF-β1 signaling in IPF is sustained, contributing to irreversible extracellular matrix deposition (Kim et al., 2018), whereas in SARS-CoV-2, it may subside with the resolution of inflammation (Alfaro et al., 2024). Still, SARS-CoV-2 fibrosis provides an opportunity to explore mechanisms involved in acute fibrotic processes, which may share some overlap with pathways observed in the chronic progression of IPF. Although the outcomes of fibrosis in these two conditions often differ, studying these shared mechanisms may inform the development of therapeutic strategies, such as TGF-β1-targeted therapies in the treatment of COVID-19 and IPF (Budi et al., 2021; P et al., 2021).

## 3 Fungal Infections in IPF

Little is presently understood about the role of fungal colonization and infection in IPF and interstitial lung disease development (Clarke et al., 2018). Implicated species requiring further investigation include *Pneumocystis jirovecii*, *Aspergillus fumigatus*, and *Candida albicans*. A comparison of fungal microbiomes between patients with IPF and controls identified *Pneumocystis jirovecii* as the predominant fungal species in two stable IPF subjects and six patients with acute exacerbations of IPF. Importantly, *P. jirovecii* was not detected in any of the 40 control patients tested, highlighting its specific association with IPF (Molyneaux et al., 2016). In addition, a study examining 18 IPF patients discovered *P. jirovecii* colonization in 27.8% of the patient population tested (Vidal et al., 2006). While the sample sizes in these studies are relatively small, the results provide an intriguing lead point for future investigation.

*Aspergillus fumigatus* is a known instigator of complications in patients with IPF; one investigation of its association with interstitial pneumonia found that 9 out of the 15 patients in the study with diagnosed pulmonary aspergillosis had IPF. The remaining 6 had non-IPF interstitial pneumonia (Kurosaki et al., 2014). The role of *A. fumigatus* is also evident in triggering an acute exacerbation of IPF in a patient with no other diagnosed medical conditions who actively took an antifibrotic agent that had previously stabilized pulmonary deterioration (Suzuki et al., 2018).

Importantly, Roudbary et al. showed that *C. albicans* was the most prevalent fungal species detected in BAL samples collected from patients with IPF. These results are intriguing because none of the individuals in this patient cohort showed any clinical indications of fungal infection for the duration of the study (Roudbary M et al., 2019). While any direct mechanism of *C. albicans* to IPF development in humans beyond the promotion of scarring via repeated lung insults has yet to be thoroughly investigated, an experiment looking at bleomycin-induced pulmonary fibrosis in mice found that intestinal overgrowth of *C. albicans* correlated with exacerbated presentations of the illness. The proposed mechanism for this aggravation is an endothelial-to-mesenchymal transition mediated by IL-17A (Fukuda et al., 2018; Yamada et al., 2023).

Finally, recent evidence has also indicated that inoculation with the fungus *Paracoccidioides brasiliensis* can induce experimental pulmonary fibrosis in mice (Franco et al., 1998; Gonzalez et al., 2008). The potential benefits of antifungal therapy in treating IPF are even less thoroughly investigated than the contribution of the mycobiome to disease pathogenesis. Still, a combination of anti-fungal itraconazole and the anti-vascular disease drug pentoxifylline therapy significantly reduced inflammation and pulmonary fibrosis in these mice (Naranjo et al., 2011).

## 4 Bacterial infections in IPF

### 4.1 Lung microbiome in pulmonary fibrosis

In recent years, altered lung microbiomes have been associated with IPF (Hewitt and Molyneaux, 2017; Puiu et al., 2024). Lung dysbiosis contributes to pulmonary inflammation by elevation of alveolar profibrotic cytokines (O’Dwyer et al., 2019) and modulation of the host immune response (Fabbrizzi et al., 2021). Pre-clinical studies of bleomycin-induced fibrosis in germ-free mice revealed that the lack of a microbiome attenuated mortality related to fibrotic injury. These results suggest that modification of lung microbiota could serve as a new approach for treating IPF (O’Dwyer et al., 2019; Fabbrizzi et al., 2021).

A Correlating Outcomes with biochemical Markers to Estimate Time-progression (COMET) study in IPF examined 55 BAL fluid samples (Han et al., 2014). DNA analysis revealed a positive association with *Staphylococcus* and *Streptococcus* genera and progression of IPF. The precise identification of bacterial species was impossible; nevertheless, the study effectively conveyed that certain *Staphylococcus* and *Streptococcus* operational taxonomic units (OTUs) are linked to worse IPF outcomes. Another study compared the bacterial burden in the BAL contents of 65 IPF patients with that of 44 controls (Molyneaux and Maher, 2014; Molyneaux et al., 2014). Not only did they find double the burden of bacteria in the BAL of the IPF patients, but they also found a strong association between patients with higher bacterial burden detected in their BAL and a decline in lung function and death. Furthermore, the study identified that the OTUs *Streptococcus* alongside Gram-negatives, *Haemophilus*, *Neisseria*, and *Veillonella* spp. were linked to IPF.

A separate analysis of BAL samples from IPF patients sought to correlate the microbiome with host immune response signaling pathways. Inflammation through fibroblast function and leukocyte phenotypes was assessed, revealing that some IPF patients exhibited changes in microbial diversity and that the lung microbiome is particularly associated with genes involved in the immune response, such as inflammation and tissue remodeling (Huang et al., 2017). Although causality was not established, the study suggests microbial influence on innate immunity and fibrosis. Other culture-independent studies have also shown an increased bacterial burden in the BAL of IPF patients (Spagnolo et al., 2019), particularly those experiencing acute exacerbation of the disease (Molyneaux et al., 2017; Invernizzi and Molyneaux, 2019). One study implicated the toll-like receptor 3 L412F polymorphism in dysregulating the lung microbiome and reducing the immune response to bacterial infection, leading to increased acute exacerbation associated-IPF death (McElroy et al., 2022).

DNA analysis reveals several distinct microbial signatures of IPF showed a characteristic abundance of *Streptococcus*, *Pseudobutyrivibrio*, and *Anaerorhabdus*. Microbial gene functionality related to ABC transporter systems (ATP synthase (ATP)-binding cassette transporters), biofilm formation, and two-component regulatory systems were more prevalent in the microbiome of IPF patients. ABC transporters are known to be involved in the efflux of antibiotics from bacterial cells (Seeger and van Veen, 2009). Since bacteria forming biofilms are encased in a matrix of extracellular polymeric substances, which render them significantly more antibiotic-resistant (Stewart and Costerton, 2001; Wu et al., 2015), it is evident that the IPF lung microbiome emphasizes key antimicrobial resistance pathways.

Beyond examining bacteria in BAL samples, a study by Kitsios et al. sought to directly analyze the microbiome of fibrotic lung tissue taken from 40 end-stage IPF patients (Kitsios et al., 2018). Contrary to previous findings, the authors found little bacterial DNA in the samples of patients with severe or acute exacerbation of IPF, therefore, comparable to controls. However, it is worth mentioning that the samples used in this study, which targeted subpleural lung tissues with significant honeycombing, originated from regions that may be unconducive to bacterial growth. Moreover, the disparity in findings may also be attributable to the end-stage sample population used in this study, compared with the early-IPF patients of most BAL studies. While the established associations between bacterial infection, progression, and severity of pulmonary fibrosis are promising, causal mechanisms have yet to be uncovered. As such, the efficacy of potential antimicrobial therapies and applications of the lung microbiome as a prognostic biomarker need to be further elucidated (Fastres et al., 2017; Ntolios et al., 2021).

### 4.2 Gram-positive bacteria

In hospitalized patients with IPF, the prevalence of bacterial pneumonia, pulmonary hypertension, and lung cancer was 9.5%, 4.6%, and 3.7%, respectively (Oda et al., 2018). Among patients with bacterial pneumonia, the two most common Gram-positive pathogens were *Streptococcus pneumoniae* (31.6%) and methicillin-resistant *Staphylococcus aureus* (MRSA) (18.4%). Respiratory comorbidities, especially bacterial pneumonia and lung cancer, influence mortality in hospitalized patients with IPF (Oda et al., 2018). Notably, a recent meta-analysis revealed that bacterial streptococcal infection occurred in 99.5% of patients with IPF (Mostafaei et al., 2021). This section will discuss the relevant research regarding bacterial infection in IPF.

*Streptococcus pneumoniae* infection has been shown to exacerbate lung fibrosis in mice *via* the release of the cytotoxin pneumolysin (Knippenberg et al., 2015); notably, fibrosis progression was mitigated when mice were given a protein-based vaccine presenting a non-cytotoxic pneumolysin derivative. This suggests that pneumolysin may, in the future, be a potential target for fibrosis treatment in human patients through a protein-based pneumococcal vaccination targeting key virulence factors like pneumolysin, which could have significant preventive effects on *S. pneumoniae-induced* fibrosis exacerbation. However, some IPF patients undergo treatment with corticosteroids and immunosuppressive agents, which have been shown to interfere with their response to the pneumococcal vaccine (Kuronuma et al., 2018). A recent study by Bormann et al. (Bormann et al., 2021) highlighted a Cox2-dependent anti-inflammatory role of prostaglandin E2 (PGE_2_) in the progression of experimental pulmonary fibrosis in mice. *Streptococcus pneumoniae*-induced IPF progression was associated with increased expression of PGE_2_, and intratracheal application of PGE_2_ worsened fibrosis in mice with AdTGF-β1-induced lung fibrosis.

A recent study reported that *Staphylococcus nepalensis* is responsible for releasing a peptide, corisin, which induces apoptosis of lung epithelial cells (D’Alessandro-Gabazza et al., 2020). The study showed that mice exposed to corisin-harboring *S. nepalensis* experienced acute exacerbation, unlike control mice, who were either untreated or infected with corisin-free bacteria. Furthermore, human IPF patients with acute exacerbation have notably higher counts of lung corisin levels than IPF patients without exacerbation.

Further, mice infected with MRSA were found to have a more difficult time fighting off infection due to fibrosis (Warheit-Niemi et al., 2022). Specifically, the authors reported that fibrosis diminished neutrophil elastase release and oxidative radical production, inhibiting intracellular killing of MRSA by neutrophils. Not only did the fibrotic mice exhibit inhibited neutrophil activity, but lung macrophages were also shown to have a reduced capacity for phagocytosis of MRSA. Thus, the study provides evidence for impaired immune response in fibrotic lungs and proposes a mechanism for why bacterial infection in individuals with IPF increases morbidity and mortality. Overall, there has been growing evidence showing an association between Gram-positive bacteria and the development of IPF and specific mechanisms by which bacteria are responsible for exacerbating the disease and hindering the immune system’s ability to fend off infection.

### 4.3 Gram-negative bacteria

Respiratory infections caused by Gram-negative bacteria in IPF patients are relatively frequent during hospitalization and are reportedly effective in predicting mortality (Yamazaki et al., 2016). In 2016, a retrospective study analyzed causative pathogens in 48 IPF patients who had been hospitalized for pulmonary infections (Yamazaki et al., 2016). The study found causative pathogens in 20/48 patients, the most common of which were *H. influenzae* (14.5%), *P. aeruginosa* (4.1%), *Moraxella* (*Branhamella) catarrhalis* (4.1%), and *Klebsiella pneumoniae* (4.1%). Intriguingly, the causative pathogens were primarily Gram-negative bacteria, in contrast to the perspective that infection with Gram-positive bacteria causes most cases of bacterial pneumonia. Moreover, the Pneumonia Severity Index (PSI) score upon admission was significantly correlated with 30-day and hospital mortality.

Besides COPD cases in adults and more pronounced cases in infants and children with cystic fibrosis, a limited connection has been made between *Bordetella pertussis* and IPF, such as a 2019 case report showing that 2 patients with IPF had been diagnosed with acute exacerbation of the disease and an acute pertussis infection (Paddock et al., 2008; Bos et al., 2011; Hashemi et al., 2015; Karamooz et al., 2018; Hirai et al., 2019). Although pertussis is preventable by vaccine, neither patient had been previously vaccinated. Thus, the study suggests further investigation into pertussis as a factor in the exacerbation of IPF and the potential use of antibiotics to treat IPF patients with infection. A 2007 study investigated the role of *Chlamydophila (Chlamydia) pneumoniae* infection in exacerbating IPF (Tomioka et al., 2007). Twenty-seven IPF patients were tested for *C. pneumoniae* antibodies, IgG, and IgA, ultimately revealing that when patients presented with an acute exacerbation of IPF, *C. pneumoniae* was not typically present.

A recent study examined the significance of macrophage scavenger receptor 1- (MSR1)-positive cells in the progression of IPF by analyzing lung transplantation tissue samples via immunohistochemistry (Zheng et al., 2021). MSR1-positive macrophages correlated with reduced lung function and poor prognostic outcomes in IPF patients (Zheng et al., 2021). MSR1 upregulation was also significantly more common in smoking patients than in non-smoking patients but also that the expression of MSR1 was significantly elevated in IPF patients infected with *K. pneumoniae*, corroborating the potential role of Gram-negative bacteria in the progression of IPF. MSR1 has been suggested to play a vital role in inflammatory, innate, and adaptive immune responses, and silica-induced fibrosis (de Winther et al., 2000; Beamer and Holian, 2005).

## 5 Role of *Haemophilus influenzae* in IPF

Among the Gram-negative bacteria meriting further investigation is *H. influenzae (H)*, as it is associated with several pulmonary afflictions. Specifically, non-typeable *H. influenzae* (*NTHi*) is a strain of *Haemophilus* bacteria that lacks a polysaccharide capsule, rendering it more difficult for the immune system to recognize and effectively defend against.

Several studies have outlined the role of *NTHi* in the development of neutrophilic asthma (Zhang et al., 2020), and others have examined the pathogenesis of *NTHi* infections in chronic suppurative lung diseases (Chatziparasidis et al., 2023). Moreover, extensive research has been done to explicitly investigate the role of *NTHi* in the pathogenesis of chronic obstructive pulmonary disease (COPD). The World Health Organization ranked COPD as the third leading cause of death in the world, with an estimated 3.23 million deaths in 2019 (Guarascio et al., 2013; World Health Organization (WHO), 2020). It is generally agreed upon that *NTHi* persists as one of the most common bacterial infections in adults with COPD and one of the major pathogens responsible for exacerbating the disease (Ahearn et al., 2017; Su et al., 2018). In patients with end-stage lung disease, *H. influenzae* was detected in the bronchi, bronchioles, damaged epithelium, and subepithelial spaces. It is thought that the near-ubiquitous presence of *H. influenzae* throughout the lung may function as a reservoir and facilitator of persistent infection. Intriguingly, the bacteria were observed to exist primarily extracellularly rather than in intracellular spaces (Moller et al., 1998). One study addressing *H. influenzae* distribution in lung tissues of patients with COPD, cystic fibrosis (CF), IPF, and other pulmonary diseases found corroborating evidence of effective and widespread invasion of pulmonary spaces. In CF, *NTHi* is proposed to colonize lungs early and contribute to disease pathogenesis by inciting airway epithelial inflammatory responses. It persists in part through the formation of biofilms, which one study observed in the BAL fluid of young, asymptomatic CF patients (Oda et al., 2018). *NTHi* may also contribute significantly to acute exacerbations in this population (Mostafaei et al., 2021). Current antibiotic treatments for CF consist primarily of *P. aeruginosa*-targeting tobramycin, colistin, and aztreonam (Knippenberg et al., 2015). Concerning IPF, a previously mentioned study found that *Haemophilus*, *Streptococcus*, *Neisseria*, and *Veillonella* species were associated with the disease by investigating the BAL of 65 patients; notably, they reported a 3.4-fold increase in *Haemophilus* in the BAL of patients with IPF in comparison to the controls (Molyneaux et al., 2014). A recurring line of reasoning has been that inflammation plays a crucial role in the pathogenesis and progression of IPF and viral and bacterial infections, which induce or worsen the condition (Bringardner et al., 2008; Homer et al., 2011). *NTHi* has been linked to the upregulation of pro-inflammatory pathways (Watanabe et al., 2004; Wilson et al., 2010; Yang et al., 2019).

*Non-typeable Haemophilus influenza* encodes several proteins that bind to plasminogen or extracellular matrix (ECM) components to damage epithelial barriers and promote persistent infection. *NTHi* enolase is particularly interesting, as it has been shown to bind primarily to plasminogen to manipulate plasmin’s proteolytic effects for tissue invasion (Osorio-Aguilar et al., 2021; Osorio-Aguilar et al., 2023). There is evidence of its ability to interact with the ECM components laminin, fibronectin, and collagen, and interaction with these proteins disrupts the regulation of cell-cell adhesion and migration. While the Haemophilus adhesive transporter domains (Haps) released from the proteolytic degradation of autotransporter adhesin and outer membrane lipoprotein (P4) can communicate with these elements, binding with vitronectin by P4-expressing NTHi is associated with the development of serum resistance (Fink et al., 2002; Su et al., 2016). Haemophilus surface protein E binds to both vitronectin and plasminogen to facilitate increased immune evasion (Barthel et al., 2012).

### 5.1 Animal studies involving *NTHi*

Several studies have revealed the importance of cytokines in NTHi. For example, interleukin-17A, a pro-inflammatory cytokine, has been demonstrated to play a role in lung fibrosis in the context of bleomycin-induced fibrosis in mice (Wilson et al., 2010). In addition, murine model of bleomycin-induced lung fibrosis has found that dysregulated lung microbiota can promote the production of interleukin-17B (IL-17B), driving disease progression (Yang et al., 2019). Other studies utilizing similar models to simulate bacterium-induced acute exacerbation of IPF by administering bleomycin to mice and then infecting them with a strain of *NTHi* (Chen et al., 2022) have revealed that *NTHi* infection can cause acute exacerbation of IPF and that the IL-17 gene is key for the progression of IPF acute exacerbation and could serve as a novel therapeutic target for treating the disease.

The decline in pulmonary function and development of fibrotic lung tissue is heavily associated with localized lung tissue inflammation (Bringardner et al., 2008; Homer et al., 2011). Several other pro-inflammatory cytokines, such as interleukin-8, interleukin-1 beta, and chemokine (C-X-C motif) ligand 1, are known to drive localized inflammation (Harada et al., 1994; Dinarello, 2011; Donahoe et al., 2015; Sawant et al., 2015). NF-kB is a transcription factor that has multiple impacts on target cells, such as regulating inflammation, inducing apoptosis, and regulating cell growth (Liu et al., 2017). *NTHi* infection has been shown to upregulate NF-kB activation with tumor necrosis factor, a pro-inflammatory cytokine (Watanabe et al., 2004). NF-kB is documented to have pro-inflammatory effects on fibroblasts, cells responsible for secreting collagen and extracellular matrix proteins, through regulation of gene transcription (Htwe et al., 2015). Fibroblast stimulation to secrete collagen and extracellular matrix proteins is one of the primary mechanisms that lead to the development of pulmonary fibrosis (Hanmandlu et al., 2022). As fibroblasts secrete increased amounts of collagen and extracellular matrix proteins, lung tissue hardens and interferes with proper gas exchange. NF-kB has also been shown to stimulate fibroblasts to secrete pro-inflammatory cytokines such as interleukin-8, macrophage inflammatory protein-1-alpha, and transforming growth factor-beta (Htwe et al., 2015). Moreover, a 2017 study found that lung fibroblasts could internalize live *NTHi*, and that fibroblasts were responsible for activating IFN-γ and IL-17A cytokine production via autologous *NTHi*-specific lung CD4^+^ T cells. This suggests that human lung fibroblasts play a crucial role in mediating T helper (Th) cell responses to bacterial infection, and specifically in conjunction with *NTHi* (Hutton et al., 2017).

Macrophages have also been shown to respond to NF-kB to secrete pro-inflammatory cytokines (Liu et al., 2017). Given that macrophages are present within alveoli, this could be another driving mechanism for localized inflammation. NF-kB is also documented to stimulate fibroblast transformation into myofibroblasts, a fibroblast state that secretes increased amounts of extracellular matrix proteins (Liu et al., 2017). Mice treated with NF-kB inhibitors were significantly protected from lung fibrosis development compared to control mice (Thakur et al., 2022). Given the wide range of effects that NF-kB has been shown to induce in various cell types, future research regarding *NTHi* and NF-kB upregulation could provide valuable information for pulmonary fibrosis pathogenesis.

Numerous mechanisms that enable *NTHi* to not only establish itself in patients with IPF, but also to maintain a persistent infection for prolonged periods of time are discussed (Murphy et al., 2004). Future studies will further reveal the specific mechanisms of *H. influenzae* in driving pathogenesis in individuals with IPF, using previously established research on related illnesses such as COPD.

### 5.2 Mechanisms involved in *NTHi* pathogenesis

*NTHi* has adapted several strategies to avoid the host immune response and maintain a persistent infection. For instance, *NTHi* uses a variety of adhesins, which allow the bacteria to attach itself to and invade host cells (Duell et al., 2016). In essence, these adhesins are essential in permitting *NTHi* to colonize primary sites, allowing further infection in secondary sites and eventually leading to the formation of biofilms and mediation of key virulence mechanisms (Duell et al., 2016). Biofilms comprise an extracellular polymeric substance inhabited by a community of bacteria. *NTHi* produces these protective biofilms to maintain a reserve of bacteria which can go on to cause subsequent infections (Gunn et al., 2016). Thus, the formation of biofilms is a key mechanism by which *NTHi* and other pathogens can sustain persistent infections in the host.

Studies have revealed that *NTHi* does not actually bind to the cell surface directly, but rather adheres to host vitronectin by means of an outer membrane protein, protein-E. The protein-E-vitronectin interaction plays a role in the adherence and invasion of *NTHi* in bronchial epithelial cells. It could serve as a potential target for novel treatments and vaccines (Ikeda et al., 2015). Lysostaphin-like metalloproteases (LytM proteins), known to facilitate cell division by affecting cleavage and membrane composition, contributed to the pathogenesis and physiology of *NTHi*. Data suggests explicitly that the component of outer membrane and cell wall physiology, the murein hydrolase activator, EnvC protein, possessing a LytM domain, might impact bacterial surface protein composition via a mechanism in which EnvC facilitates the transport of periplasmic chaperones to the outer membrane. This is further supported by studies showing that an *NTHi envC-*defective strain has diminished capacity to adhere to epithelial cells and form biofilm, while also displaying a reduced resistance to the immune system (Ercoli et al., 2015).

Although *NTHi* is widely considered an extracellular pathogen, *NTHi* has also been spotted residing intracellularly (St Geme and Falkow, 1990). For example, *NTHi* lipooligosaccharide interacts with the platelet-activating factor receptor, enabling *NTHi* to adhere to and infiltrate human bronchial epithelial cells (Swords et al., 2000). Morey et al. proposed a unique mechanism by which *NTHi* can invade the airway epithelium and reside intracellularly, involving the assembly of microtubules, integrity of lipid rafts, and activation of phosphatidylinositol 3-kinase (PI3K) signaling (Morey et al., 2011). *NTHi* were found to primarily reside in acidic sub-cellular vacuoles with late endosome features, existing in a metabolically active yet non-replicative state. This *NTHi*-containing vacuole co-localizes with LysoTracker, lysosome-associated membrane protein 1 (LAMP1), LAMP2, CD63, and Rab7 and does not possess Golgi- or autophagy-related markers. It may modulate epigenetic changes like many known bacterial nucleomodulins or by altering host endosomal proteins mechanisms to evade host immune response and gain intracellular persistence (Morey et al., 2011; Cohen et al., 2013; Denzer et al., 2020; Fol et al., 2020; Wrede et al., 2023).

**Figure 2:**
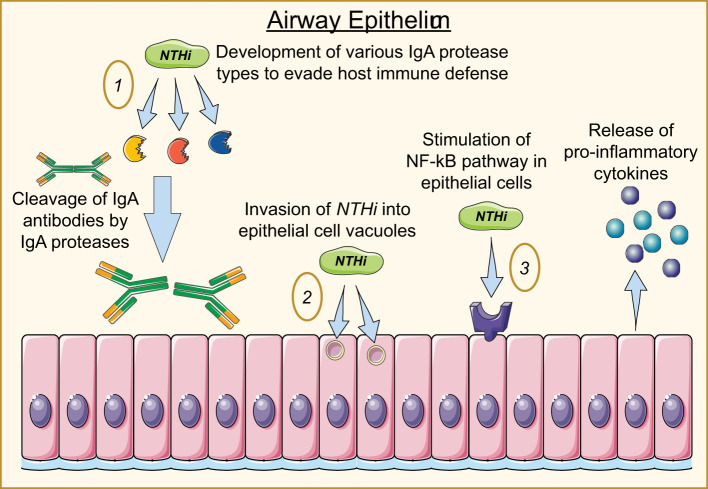
Mechanisms of *NTHi* invasion into airway epithelial cells. 1) *NTHi* produces three IgA protease types responsible for destroying IgA antibodies and enabling the bacterium to invade and persist within the host cell. 2) *NTHi* enters the cell through endocytic mechanisms, hiding in vacuoles to avoid degradation. 3) *NTHi* stimulates the NF-kB pathway, producing cytokine secretion while promoting a series of pro-inflammatory responses from fibroblasts and macrophages.

Further investigation has suggested that *NTHi* strategically positions itself intracellularly to avoid immune responses and maintain a persistent infection, and IgA proteases are essential to *NTHi*’s ability to do this. Epithelium and airway mucous membranes contain IgA as a part of the innate immune system to defend against pathogens; in the upper respiratory tract, IgA1 is the dominant form (Salvi and Holgate, 1999). *H. influenzae* is documented to produce three types of IgA1 proteases that can cleave the heavy chain of IgA1 antibodies and render the Fc portion dysfunctional. While most capsulated strains of *H. influenzae* produce only one of the three IgA1 proteases, *NTHi* strains produce any of the three types (Foxwell et al., 1998). Each of the three proteases play distinct roles in the pathogenesis of *NTHi.* While IgA protease is necessary for *NTHi* invasion, IgA proteases B1 and B2 are essential to the intracellular persistence of *NTHi* (Murphy et al., 2017). Not only do IgA proteases serve to protect *NTHi* from mucosal immunity by cleaving human IgA1, but they have also been found to facilitate intracellular survival by cleavage of LAMP1 at each stage of the endolysosomal pathway, including the plasma membrane, early and late endosome, and lysosome stages. In doing so, IgA proteases B1 and B2 facilitate *NTHi*’s ability to evade the endo-lysosomal pathway, though *NTHi* is ultimately destroyed in lysosomes after variable durations of intracellular survival (Clementi et al., 2014). NTHi has also been documented invading epithelial cells and residing in membrane-bound vacuoles inside epithelial cells (Foxwell et al., 1998). The mechanisms of NTHi pathogenesis are depicted in **Figure 2**.

To ensure that *NTHi* persists in various hostile COPD environments, the bacteria use several mechanisms for genetic variation, including antigenic variation (Sethi et al., 2004), phase variation (Murphy et al., 2004), and epigenetic variation (Atack et al., 2015). Additionally, *NTHi* must adapt specific metabolic pathways to take in necessary nutrients. For instance, ferric uptake regulator (*Fur*)-regulated genes in *NTHi* modulate iron usage within the cell, ultimately facilitating more severe and persistent infections (Duell et al., 2016).

Because iron is in limited supply within the host, *NTHi* sequesters iron to ensure its survival. Recent dual RNA-Seq studies have further elucidated these metabolic adaptations, revealing that *NTHi* upregulates iron acquisition pathways and modifies metabolic processes to survive under intracellular conditions (Baddal et al., 2015). These studies also show that *NTHi* dynamically adjusts its transcriptional responses in real time, responding to the intracellular environment to maximize survival. Concurrently, *NTHi* modulates host immune responses, suppressing inflammatory signaling pathways to evade detection and maintain a permissive intracellular environment (Ackland et al., 2021). This dual role of metabolic adaptation and immune modulation enables *NTHi* to persist intracellularly, protecting itself from immune clearance and ensuring long-term colonization of the host.

Using the mechanisms outlined above, *NTHi* can establish a persistent infection in the lower airways, lasting months to years. Moreover, this wide range of unique virulence mechanisms allows *NTHi* to persist in its host and exacerbate pulmonary diseases, including but not limited to IPF. Therefore, *NTHi* is particularly interesting when evaluating possible concurrence and associations with IPF. Mechanisms discussed above could play a role in the progression of IPF in patients infected with *H. influenzae.*

### 5.3 Limitations of bleomycin model of pulmonary fibrosis in preclinical studies

Most preclinical lung fibrosis studies widely use the chemotherapy drug bleomycin sulfate, which is intratracheally administered in rats and mice to induce an inflammatory response progressing into lung fibrosis that mimics the pathology of IPF and airway remodeling (Polosukhin et al., 2012; Tanjore et al., 2013). Bleomycin-induced pulmonary fibrosis is highly reproducible, with key molecular pathways triggered by reactive oxygen species and inflammation that leads to excessive collagen deposition and fibrosis (Degryse et al., 2011; Polosukhin et al., 2012; Tanjore et al., 2013; Moss et al., 2022). The rodent models are also employed to examine the *in vivo* efficacy of drugs for preventive and therapeutic potentials for IPF treatment (Kolb et al., 2020). While these models capture many qualitative histological aspects of IPF, they may not completely replicate all the complexities of the human disease, given the chronic nature of the disease in IPF patients. On a histologic level, bleomycin-induced fibrosis lacks the basal and subpleural predominance characteristic of IPF and involves more limited alveolar epithelial cell remodeling (Moss et al., 2022). However, repetitive bleomycin dosing and dosing of aged mice show promising results that more closely approximate non-reversible fibrotic phenotypes and classic histologic presentations of IPF (Degryse et al., 2010; Weckerle et al., 2023). Interestingly, positron-emission tomography-computed tomographic scanning was employed in bleomycin-induced mice to closely mimic the diagnostic methods of IPF and evaluate pathologic changes with different modes of bleomycin administration. It was shown that repeated intravenous bleomycin delivery for seven consecutive days matched closer to the early pathologic features of idiopathic pulmonary interstitial fibrosis (Sgalla et al., 2016; Comes et al., 2020; Gul et al., 2023).

## 6 Antibiotics and other therapeutics in the treatment of IPF

### 6.1 Completed clinical trials

#### 6.1.1 Antivirals

Numerous studies have evaluated the use of antibiotics to help treat IPF. Despite the prevalence of IPF worldwide, our understanding of how persistent infection plays into the pathogenesis and advancement of the illness is still incomplete. Much of the research on viral infections has centered on the herpesviruses, particularly EBV. Although the presence of EBV in IPF patients is valid, little research has been done to investigate the potential role of other viruses, such as human metapneumovirus, influenza virus, and coronaviruses. Exploring the comorbidity of other viruses with IPF is beneficial, especially considering that evidence points to antiviral regimens as a valid approach to treating the disease. Ganciclovir, an antiviral medication, successfully mitigates the progression of IPF (Folcik et al., 2014), and concurrent treatment with pirfenidone, the anti-fibrotic drug, and valganciclovir, the prodrug of ganciclovir, was well tolerated in patients with IPF and could serve as a viable treatment (Study# 1, **Table 1**) (Blackwell et al., 2021). Azithromycin, in addition to its antimicrobial properties, has been found to possess anti-inflammatory effects, modulating the immune response by reducing the production of inflammatory cytokines and chemokines (Gao et al., 2010). Like other macrolide antibiotics, azithromycin can also penetrate cells and accumulate intracellularly, which is particularly relevant in treating intracellular bacterial infections such as those discussed for *NTHi*. In fact, Azithromycin’s capacity for intracellular activity may contribute to its anti-inflammatory properties, as it can modulate immune responses within cells (Munic et al., 2011). A retrospective study assessing the effects of azithromycin found that the mortality rate in IPF patients treated with azithromycin (26%) was significantly lower than that of those treated with fluoroquinolones (70%) (Kawamura et al., 2017). A more recent retrospective study investigated how IPF patients responded to prophylactic azithromycin. This study found that hospital admissions and antibiotic courses were significantly lower in the 12 months following the use of prophylactic azithromycin compared to the no-prophylactic treatment group (Macaluso et al., 2019). However, a 2021 randomized controlled trial showed no substantial benefit from treatment with low-dose azithromycin for chronic cough in patients with IPF (Study #3, **Table 1**) (Guler et al., 2021). The most prominent and recent SARS-COV2-associated lung disease, COVID-19, shares common risk factors with IPF. These risk factors include age-associated inflammation and metabolic syndromes, particularly in male patients.

**Table 1:** List of completed clinical trials conducted in IPF patients.

Study No.	Study Title	Conditions	Interventions	Clinical Trial Number	Current Status	Results
1	A Pilot Trial of Herpesvirus Treatment in Idiopathic Pulmonary Fibrosis (IPF)	Idiopathic Pulmonary Fibrosis	Drug: Valganciclovir Other: Placebo	NCT02871401	Completed	Safe and well tolerated
2	The Efficacy and Mechanism Evaluation of Treating Idiopathic Pulmonary Fibrosis with the Addition of Co-trimoxazole (EME-TIPAC)	Idiopathic Pulmonary Fibrosis	Drug: Co-trimoxazole	EUDRACT 2014-004058-32	Completed	Lack of efficacy
3	Azithromycin in Idiopathic Pulmonary Fibrosis	Idiopathic Pulmonary Fibrosis, Cough	Drug: Azithromycin Other: Placebo	NCT02173145	Completed	Lack of efficacy
4	Diffuse Fibrotic Lung Disease	Lung Diseases, Pulmonary Fibrosis, Sarcoidosis	Drug: Prednisone, Cyclophosphamide, Dapsone	NCT00000596	Completed	May improve outcomes
5	Combined PEX, Rituximab and Steroids in Acute Idiopathic Pulmonary Fibrosis Exacerbations	Idiopathic Pulmonary Fibrosis	Drug: Combined Plasma Exchange (PEX), Rituximab, and Corticosteroids	NCT01266317	Completed	May improve outcomes
6	Cyclosporine in Patients With Moderate COVID-19	COVID-19 Acute Respiratory Distress Syndrome, Cytokine Release Syndrome	Drug: Cyclosporine A	NCT04412785	Completed	Safe, may improve outcome
7	Safety and Tolerability Study in Subjects With Idiopathic Pulmonary Fibrosis (IPF)	Idiopathic Pulmonary Fibrosis	Drug: Tipelukast Other: Placebo	NCT02503657	Completed	Safe and well tolerated
8	STX-100 in Patients With Idiopathic Pulmonary Fibrosis (IPF)	Idiopathic Pulmonary Fibrosis	Drug: BG00011 Other: Placebo	NCT01371305	Completed	Safe and well tolerated
9	A Phase 2 Study to Evaluate the Safety and Tolerability of PBI-4050 in Patients With Idiopathic Pulmonary Fibrosis (IPF)	Idiopathic Pulmonary Fibrosis	Drug: PBI-4050	NCT02538536	Completed	Safe and well tolerated
10	A Study to Test the Efficacy and Safety of Inhaled GB0139 in Subjects With Idiopathic Pulmonary Fibrosis (IPF)	Idiopathic Pulmonary Fibrosis	Drug: GB0139 Other: Placebo	NCT03832946	Completed	Lack of efficacy

Thus, available antifibrotic therapies could help prevent severe COVID-19 in patients with IPF or treat severe COVID-19 in patients without IPF (George et al., 2020). The safety and effectiveness of cyclosporin A, an immunosuppressor, have been tested in recent clinical trials in COVID-19 patients and were demonstrated to be effective with a significant reduction in inflammatory cytokines and chemokines, including CXCL10 in patients requiring oxygen support (Study #6, **Table 1**). The administration of cyclosporin was reportedly safe and could be feasible as an adjunct therapy in resource-limited healthcare settings (Blumberg et al., 2022). However, more research is needed to evaluate its efficacy beyond acute management for patients with long-term COVID-19 lung diseases.

#### 6.1.2 Antibacterials and immunosuppressants

Concerning antibacterial agents, EudraCT (European Union Drug Regulating Authorities Clinical Trials) clinical trials studied Co-trimoxazole and two antibiotics, trimethoprim and sulfamethoxazole, in IPF patients (Study #2, **Table 1**). Addressing the harmful accumulation of neutrophils within alveoli, which occurs in patients with IPF, a study that administered combined IV doses of methylprednisolone and prednisone found that the use of intermittent, high-dose “pulse” corticosteroid injections might help mitigate the chronic effect of neutrophil aggregation on alveolitis in IPF (Study #4, **Table 1**) (Keogh et al., 1983).

Furthermore, the combination of therapeutic plasma exchanges, rituximab, and corticosteroids (Study #5, **Table 1**) may benefit patients with severe acute exacerbation of IPF. These studies warrant further investigation into the role of autoantibodies in the progression of IPF and the efficacy of autoantibody reduction treatments (Donahoe et al., 2015). Safety and tolerability tests of tipelukast, a sulfidopeptide leukotriene receptor antagonist, which exerts anti-inflammatory and anti-fibrotic effects (Study #7, **Table 1**) did not meet the primary endpoint, although safe and well-tolerated. BG00011, a monoclonal anti- αvβ6 integrin antibody (Study #8, **Table 1**), was demonstrated to be safe and well-tolerated. PBI-4050 (Study #9, **Table 1**), a small molecule inhibitor of fibrosis, was found to be safe and well-tolerated at lower doses. GB0139 (Study #10, **Table 1**) showed neither worsening nor improvement in patients with IPF. However, further research is required to determine the utility of these drugs in treating pulmonary fibrosis.

### 6.2 Terminated and ongoing clinical trials in the treatment of IPF

A randomized, placebo-controlled trial was carried out to determine the effects of co-trimoxazole in patients with IPF (Shulgina et al., 2013). Despite a previous trial indicating an improvement in forced vital capacity (FVC) (Varney et al., 2008), this extensive study showed no significant differences in FVC between the co-trimoxazole-treated patients versus the placebo groups who received co-trimoxazole treatment at four and half months later timepoint after decoding the study. There was, however, a decrease in overall mortality rates across the two groups and a reduction in subsequent respiratory tract infections, suggesting that delayed treatment could also help recuperate with a significantly improved MRC5 Point Dyspnoea Score (Study #1, **Table 2**). A 2013 study designed to evaluate the effect of co-trimoxazole on IPF was terminated due to changes in standards of care (Study #2, **Table 2**), while results from another clinical trial did not support the use of the drug to treat patients with moderate to severe IPF (Wilson et al., 2020). Similarly, a 2021 study investigating the clinical performance of antimicrobial therapy in IPF found that the use of co-trimoxazole or doxycycline did not significantly improve the time to non-elective respiratory hospitalization or death, concluding that these antibiotics are ineffective in treating the underlying disease (Anstrom et al., 2020; Martinez et al., 2021). Another study evaluating the efficacy of minocycline as a treatment for IPF was conducted, although the results were inconclusive (Study #3, **Table 2**). A trial assessing simtuzumab was terminated due to low efficacy (Study #4, **Table 2**). Similarly, a clinical trial in 2011 was terminated upon finding that ambrisentan was ineffective in treating patients with IPF and may even worsen the condition (Raghu et al., 2013).

**Table 2:** List of terminated and ongoing clinical trials conducted in IPF patients.

Study No.	Study Title	Conditions	Interventions	Clinical Trial Number	Current Status	Results
1	CleanUP IPF for the Pulmonary Trials Cooperative (CleanUp-IPF)	Idiopathic Pulmonary Fibrosis	Drug: Co-trimoxazole or Doxycycline Other: No Intervention: Standard of Care	NCT02759120	Terminated	Lack of efficacy
2	Study to Test the Validity of the Treatment of Idiopathic Pulmonary Fibrosis With Cotrimoxazole (TriSulfa-FPI)	Idiopathic Pulmonary Fibrosis	Drug: Cotrimoxazole Other: Placebo	NCT01777737	Terminated	Changes in standard of care
3	Minocycline Therapy for Lung Scarring in Patients With Idiopathic Pulmonary Fibrosis - a Pilot Study	Idiopathic Pulmonary Fibrosis	Drug: minocycline	NCT00203697	Unknown	N/A
4	Long-Term Safety Study of GS-6624 in Adults With Idiopathic Pulmonary Fibrosis (IPF) (ATLAS)	Idiopathic Pulmonary Fibrosis	Drug: simtuzumab	NCT01759511	Terminated	Lack of efficacy
5	Randomized, Placebo-Controlled Study to Evaluate Safety and Effectiveness of Ambrisentan in IPF (ARTEMIS-IPF)	Idiopathic Pulmonary Fibrosis	Drug: Ambrisentan Other: Placebo	NCT00768300	Terminated	Lack of efficacy
6	A Study of PMG1015 Injection in Idiopathic Pulmonary Fibrosis Subjects	Idiopathic Pulmonary Fibrosis	Drug: PMG1015 Other: Placebo	NCT05895565	Recruiting	N/A
7	Phase I Study to Assess Safety, Tolerability, PK and PD of AGMB-447 in Healthy Participants and Participants With IPF	Idiopathic Pulmonary Fibrosis	Drug: AGMB-447 Other: Placebo	NCT06181370	Recruiting	N/A
8	Study Evaluating INS018_055 Administered Orally to Subjects With Idiopathic Pulmonary Fibrosis	Idiopathic Pulmonary Fibrosis	Drug: INS018_055 Other: Placebo	NCT05975983	Recruiting	N/A
9	Tolerability, Pharmacokinetics and Efficacy of ZSP1603 in Patients With Idiopathic Pulmonary Fibrosis (IPF)	Idiopathic Pulmonary Fibrosis	Drug: ZSP1603 Other: Placebo	NCT05119972	Recruiting	N/A
10	Study to Assess the Safety, Pharmacokinetics, Pharmacodynamics and Clinical Activity of RXC007 in Idiopathic Pulmonary Fibrosis	Idiopathic Pulmonary Fibrosis, Fibrosis	Drug: RXC007 Other: Placebo	NCT05570058	Active, not recruiting	N/A
11	A Study to Investigate Leramistat in Patients With IPF	Idiopathic Pulmonary Fibrosis	Drug: Leramistat Other: Placebo	NCT05951296	Recruiting	N/A
12	LYT-100 in Patients With Idiopathic Pulmonary Fibrosis (IPF) (ELEVATE)	Idiopathic Pulmonary Fibrosis	Drug: Pirfenidone, Deupirfenidone Other: Placebo	NCT05321420	Active, not recruiting	N/A
13	A Study to Evaluate LTI-03 in Newly Diagnosed Idiopathic Pulmonary Fibrosis (IPF) Patients	Idiopathic Pulmonary Fibrosis	Drug: LTI-03 Other: Placebo	NCT05954988	Active, not recruiting	N/A
14	Prospective Treatment Efficacy in IPF Using Genotype for Nac Selection (PRECISIONS) Trial (PRECISIONS)	Idiopathic Pulmonary Fibrosis	Drug: N-acetyl cysteine Other: Placebo	NCT04300920	Active, not recruiting	N/A
15	Randomized, Double-blind Study of Efficacy and Safety of Bexotegrast (PLN-74809) for Idiopathic Pulmonary Fibrosis	Idiopathic Pulmonary Fibrosis	Drug: PLN-74809 Other: Placebo	NCT06097260	Recruiting	N/A
16	Phase I/ IIa Clinical Trial of Human Umbilical Cord Mesenchymal Stem Cell Injection in the Treatment of Idiopathic Pulmonary Fibrosis (IPF)	Idiopathic Pulmonary Fibrosis	Drug: Human umbilical cord mesenchymal stem cell injection	NCT05468502	Recruiting	N/A

An initial phase I trial involving healthy participants revealed that INS018_055, an AI-generated drug designed to treat IPF, was both safe and tolerable (Ren et al., 2024). The drug is currently being evaluated in IPF patients, with promising potential for treatment (Study #8, **Table 2**). Several other drugs that are currently being evaluated as potential treatments for IPF, as listed in **Table 2**, include PMG1015, AGMB-447, ZSP1603, RXC007, Leramistat, Pirfenidone and Deupirfenidone, LTI-03, N-acetyl cysteine, and PLN-74809 (Studies# 9-14, **Table 2**). Interestingly, a crucial ongoing clinical trial is currently recruiting patients to evaluate the utility of human umbilical cord MSC injections in treating IPF (Study #16, **Table 2**). In a preclinical model, Moodley et al. showed that human umbilical cord mesenchymal stem cells (MSCs) could reduce fibrosis in a bleomycin-induced mouse lung injury model (Moodley et al., 2009). Since then, several studies have gone on to show the clinical safety of administering autologous adipose-derived stem cells residing in the stromal vascular fraction (SVF) of white adipose tissue (Tzouvelekis et al., 2013), intravenous allogenic human placenta-derived MSCs (Chambers et al., 2014), and a single infusion of allogenic human bone marrow-derived MSCs in patients with moderate to severe IPF (Glassberg et al., 2017).

### 6.3 Clinical studies exploring diagnostic methods

The discovery of novel diagnostic measures to better mark the presence and progression of IPF remains an essential goal for future research. In addition, investigating the efficacy of antibiotics and other therapeutics could enhance the quality and number of treatment options available for patients with IPF. A 2018 pilot study utilized ultra-high-performance liquid chromatography and high-resolution mass spectrometry to search for distinct IPF metabolic profiles in exhaled breath condensate (EBC) samples (Rindlisbacher et al., 2017). Although this approach requires replication across larger sample sizes to confirm diagnostic utility, investigators discovered preliminary evidence for differential regulation of 26 metabolic features between IPF patients and health controls. Assuming such findings are not exclusive to patients with advanced disease, early IPF detection via altered metabolic activity detection could facilitate more prompt treatment and improve patient outcomes. Another study used similar methods to perform serum metabolic profiling of patients with IPF, identifying a lysophosphatidylcholine as a potential biomarker (Rindlisbacher et al., 2018). Lysophosphatidylcholine is a precursor to lysophosphatidic acid, an established mediator of fibrotic development. An ongoing project which began in 2005 aims to identify and characterize genetic and biological markers of IPF (Study #1, **Table 3**). After accessing and analyzing data collected from patients in this study as well as from the ARTEMIS-IPF trial (Study #5, **Table 2**), researchers found an association between elevated levels of lysyl oxidase-like 2, which facilitates the cross-linking of collagen within the pathological stroma, and increased risk for IPF disease progression and mortality (Chien et al., 2014). Successful identification of reliable biomarkers would supplement and reinforce existing diagnostic methods for IPF and enhance our capacity to monitor drug effects. More studies should be conducted to improve our understanding and treatment of the disease. Furthermore, a closer look into specific demographics disproportionately affected by IPF, such as that performed by an observational study conducted in Spain (Study #2, **Table 3**), could offer insight into how IPF manifests within societies. Using demographic information to identify individuals at higher risk of IPF could help direct screening efforts and promote early identification of those with the disease.

**Table 3:** List of clinical studies exploring diagnostic methods.

Study No.	Study Title	Conditions	Interventions	Clinical Trial Number	Current Status	Results
1	Genomic and Proteomic Analysis of Disease Progression in Idiopathic Pulmonary Fibrosis (GAP)	Idiopathic Pulmonary Fibrosis	N/A	NCT00373841	Recruiting	N/A
2	OASIS-IPF (Idiopathic Pulmonary Fibrosis) Study	Idiopathic Pulmonary Fibrosis	N/A	NCT03386994	Completed	Observational correlations

## 7 RNA-based therapeutics

Over recent years, RNA has gained significant traction as a potential therapeutic due to its ability to express proteins through mRNA, silence genes through siRNA, and even edit genes through the CRISPR/Cas9 system (Coutinho et al., 2019). Chemically modified cystic fibrosis transmembrane conductance regulator (CFTR) mRNA was used to treat cystic fibrosis in CFTR knockout mice. These treated mice recovered up to 55% of the chloride levels seen in wild-type mice, and restored activity lasted a minimum of 2 weeks (Robinson et al., 2018). mRNA vaccines encoding the HA protein from the influenza A H1N1 virus can induce robust humoral and cellular immune responses, effectively shielding mice from the viral infection (Zhuang et al., 2020). Most recently, Pfizer-BioNTech and Moderna developed two mRNA-based COVID-19 vaccines, effectively mitigating the spread and severity of the emerging coronavirus disease. Both vaccines utilized lipid nanoparticle (LNP)-based delivery systems to introduce the therapeutic effectively (Buschmann et al., 2021). This method could be effective in treating other diseases like IPF.

Lipid nanoparticles (LNPs) facilitate internalization, endosomal distribution, and subsequent delivery of mRNA-based therapeutics into the cytosol (Hou et al., 2021). The optimal function of LNPs involves transporting mRNA to endosomal compartments for release into the cytoplasm or even degradation, so long as normal endosomal functions are not disrupted. The distribution of LNP-mRNA formulations was significantly higher in early endosomes, with a greater fraction of highly effective LNP formulations residing in early and recycling endosomes, indicating the therapeutic advantage of LNP-mRNA delivery (Paramasivam et al., 2022). Utilizing mathematical modeling, Paramasivam et al. found that recycling endosomal compartments was most effective in facilitating mRNA escape. However, continuous uptake of LNP formulations interferes with endosomal acidification, inhibiting the maturation of early endosomes into late endosomes and resulting in mRNA accumulation in large endosomal compartments associated with ineffective delivery. Thus, the release of mRNA was found to predominantly occur from small-sized early endosomes and recycling tubular endosomes (Paramasivam et al., 2022). Ultimately, it was revealed that progressive accumulation of LNPs in large endosomes interferes with endosomal maturation and impairs effective mRNA delivery. These cytotoxic effects can be circumvented by developing LNP formulations that can be uniformly distributed across endosomal compartments or selectively transported to recycling tubules for efficient mRNA escape. While addressing these obstacles may improve the efficiency of LNPs, this is only one avenue to optimize the delivery of mRNA therapies.

### 7.1 LNP-RNA formulations tested in preclinical studies on pulmonary fibrosis

In the mouse model of fibrosis, administration of mannose-incorporated LNPs carrying siRNAs, which down-regulate the EMT-associated protein, G2 and S phase-expressed protein 1(GTSE1), resulted in a notable reduction in collagen accumulation and EMT-related proteins and functional recovery from pulmonary fibrosis (Jin et al., 2023). Moreover, intratracheal administration of luciferase mRNA LNPs led to targeted lung accumulation in bleomycin-induced lung fibrosis. Bioluminescence was detected in the lungs as early as 2 hours post-delivery and persisted for 48 hours, with LNPs associating with AEC2 cells and fibroblasts *in vivo* (Massaro et al., 2023). These studies underscore the potential of LNPs as a promising approach for treating pulmonary fibrosis by facilitating targeted delivery of RNA therapeutics in the lungs. More recently, inhalable LNPs delivering dual mRNAs were shown to restore AT2 cell function and promote alveolar regeneration in IPF in bleomycin-induced lung fibrosis. These LNPs corrected mitochondrial dysfunction, prevented premature AT2 cell senescence, and halted pathological epithelial remodeling and fibroblast activation, facilitating alveolar regeneration (Wang et al., 2024). Using the LNP-mRNA system, this study demonstrated robust protein expression in lung epithelial cells, effectively reversing alveolar collapse and improving survival in fibrosis mice, which further supports using LNP-mRNAs as potential treatments against IPF.

### 7.2 LNP-RNA formulations tested in preclinical studies on lung infections

Just as LNP-mRNAs may target the fibrotic pathways of IPF directly, *in vitro* and *in vivo* mRNA transfections have been previously shown to enhance epithelial resistance to invading bacteria by increasing local antimicrobial peptide levels (Zou et al., 2013; Maruggi et al., 2017). Nevertheless, two mRNA vaccines, encoding PcrV and OprF-I, both components of *Pseudomonas aeruginosa*, were delivered via LNPs and tested *in-vivo* infection models. Both vaccines elicited strong immune responses and reduced bacterial burden and inflammation in infection models. While mRNA-PcrV showed superior antigen-specific responses and higher survival rates against PA strains compared to mRNA-OprF-I, the combined mRNA vaccine demonstrated the highest survival rate and outperformed protein vaccines (Wang et al., 2024).

More recently, a group created LNP-mRNAs to enhance anti-MRSA (multidrug-resistant *Staphylococcus aureus*) immunity via *in situ* programming of macrophages. The nanoparticles delivered MRSA-targeted chimeric antigen receptor (CAR) mRNA to enhance macrophages’ ability to recognize and attack MRSA and CASP11 siRNA, inhibiting a key MRSA intracellular evasion mechanism. This resulted in the creation of CAR-macrophages with enhanced bactericidal ability, which could efficiently phagocytose and destroy MRSA intracellularly, overcoming the bacterium’s immune evasion (Tang et al., 2024). Thus, these findings highlight the potential of LNP-mRNA formulations in patients, combatting pathogens like *Pseudomonas aeruginosa* and MRSA, and infection-related mechanisms of IPF pathogenesis.

**Figure 3:**
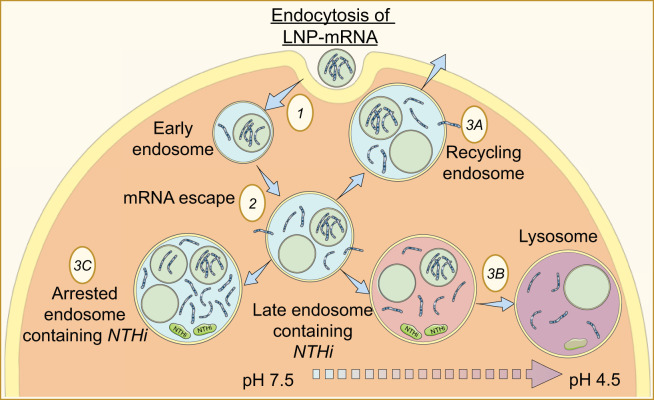
Proposed Model of lipid nanoparticle delivery of mRNA to *NTHi-*containing endosomes. (1) LNP-mRNAs enter the cell via endocytosis and are transported to early endosomes. (2) As the endosomal lumen acidifies, mRNA escapes first from the LNP, and then from the endosome altogether and into the cytoplasm. Escape of mRNA is most prominent in small early endosomes and recycling endosomes. From here, LNP-mRNAs follow one of three pathways: (3A) LNP-mRNAs are sorted to recycling endosomes (most favorable for mRNA escape), where they are transported back to the plasma membrane; (3B) LNP-mRNAs progress through the endocytotic pathway, carried by late endosomes to lysosomes where escape is poor, and they are ultimately degraded; (3C) LNP-mRNAs accumulate in large early endosomes where maturation is arrested inhibited, and delivery of mRNA is nonproductive.

The use of LNPs in delivering targeted mRNA therapies shows promise in treating other bacterial infections, which could provide new avenues for addressing infection-related mechanisms of IPF. Further pre-clinical and clinical research is needed to explore the efficacy of LNP-mRNA therapies with other intracellular pathogens such as *NTHi*. Thus, as we continue to uncover new strategies for creating high-efficiency LNPs, our understanding of how to treat illnesses with mRNA-based therapeutics will improve dramatically. To that end, the use of targeted LNP-mRNAs could drastically improve the treatment of patients with IPF. Therapeutic strategies using LNP-mRNAs in intracellular persistent infections such as *NTHi* in ILDs, including IPF are depicted in **Figure 3**.

## 8 Conclusion and future insights

Via the internalization mechanism of LNPs outlined above, mRNA-based therapeutics could surpass the limitations of traditional antibiotics and be transported intracellularly, penetrating the plasma membrane and interacting with infected compartments such as *NTHi-*containing endosomes. Through LNPs, medicines could be employed against evasive and persistent intracellular infections that would otherwise be inaccessible. With improving LNP delivery, mRNA vaccines could be used to treat and prevent viral and bacterial infections in IPF patients. Furthermore, self-amplifying mRNA vaccines have shown promising results in fending off pathogens (Maruggi et al., 2017). They could offer potential solutions to the limitations of conventional mRNA vaccines (Bloom et al., 2021), namely, antigen expression and robust elicitation of host immune response that relies on the number of mRNA transcripts delivered.

Despite the growing evidence that bacterial infection may play a key role in the progression and exacerbation of IPF, further investigation is still needed to uncover the precise mechanisms at work. Studies have shown that Gram-positive Staphylococcus, releases a peptide associated with the exacerbation of IPF and is also responsible for altering the host’s ability to fight infection (D’Alessandro-Gabazza et al., 2020). Likewise, *Streptococcus* has been shown to release a cytotoxin associated with the progression of lung fibrosis (Knippenberg et al., 2015). The role of Gram-negative bacteria in IPF is less understood. For example, several studies have drawn connections between the presence of *H. influenzae* and *K. pneumoniae* and the worsening of IPF (Yamazaki et al., 2016). Yet, more mechanistic insight is needed regarding bacterial-induced IPF pathogenesis. Further investigation into the pathogenic role of intracellular *NTHi* in IPF disease states could bolster our understanding of how the condition progresses over time and offer new insight into potential therapeutic approaches, as evidenced by the numerous promising studies evaluating antibiotic treatment in patients with IPF. Specifically, more efforts should be made to elucidate the role of *NTHi* in the pathogenesis and progression of IPF, given its distinct capacity to persist in the airways of COPD patients. Investigation in these areas would enhance our functional knowledge of IPF. It may provide researchers and clinicians with the theoretical basis for developing novel treatments to combat the devastating effects of IPF. Ultimately, a better grasp of the fundamental mechanisms at work will pave the way for novel treatments and an improved prognosis for IPF in the coming years.
